# InformedTogether: Usability Evaluation of a Web-Based Decision Aid to Facilitate Shared Advance Care Planning for Severe Chronic Obstructive Pulmonary Disease

**DOI:** 10.2196/humanfactors.3842

**Published:** 2015-02-25

**Authors:** Lauren M Uhler, Rafael E Pérez Figueroa, Mark Dickson, Lauren McCullagh, Andre Kushniruk, Helen Monkman, Holly O Witteman, Negin Hajizadeh

**Affiliations:** ^1^ Department of Medicine Hofstra North Shore LIJ School of Medicine North Shore LIJ Health System Manhasset, NY United States; ^2^ Pediatrics and Population and Family Health College of Physicians and Surgeons and Mailman School of Public Health Columbia University Medical Center New York, NY United States; ^3^ International Family AIDS Global Health Program (IFAP) Columbia University Medical Center New York, NY United States; ^4^ Sitesteaders Development Ypsilanti, MI United States; ^5^ School of Health Information Science University of Victoria Victoria, BC Canada; ^6^ Department of Family and Emergency Medicine Laval University Quebec City, QC Canada; ^7^ Office of Education and Continuing Professional Development Faculty of Medicine Laval University Quebec City, QC Canada; ^8^ Research Centre of the CHU de Québec Quebec City, QC Canada

**Keywords:** usability testing, decision aid, shared decision making, COPD, advance care planning

## Abstract

**Background:**

Advance care planning may help patients receive treatments that better align with their goals for care. We developed a Web-based decision aid called InformedTogether to facilitate shared advance care planning between chronic obstructive pulmonary disease (COPD) patients and their doctors.

**Objective:**

Our objective was to assess the usability of the InformedTogether decision aid, including whether users could interact with the decision aid to engage in tasks required for shared decision making, whether users found the decision aid acceptable, and implications for redesign.

**Methods:**

We conducted an observational study with 15 patients and 8 doctors at two ethnically and socioeconomically diverse outpatient clinics. Data included quantitative and qualitative observations of patients and doctors using the decision aid on tablet or laptop computers and data from semistructured interviews. Patients were shown the decision aid by a researcher acting as the doctor. Pulmonary doctors were observed using the decision aid independently and asked to think aloud (ie, verbalize their thoughts). A thematic analysis was implemented to explore key issues related to decision aid usability.

**Results:**

Although patients and doctors found InformedTogether acceptable and would recommend that doctors use the decision aid with COPD patients, many patients had difficulty understanding the icon arrays that were used to communicate estimated prognoses and could not articulate the definitions of the two treatment choices—Full Code and Do Not Resuscitate (DNR). Minor usability problems regarding content, links, layout, and consistency were also identified and corresponding recommendations were outlined. In particular, participants suggested including more information about potential changes in quality of life resulting from the alternative advance directives. Some doctor participants thought the decision aid was too long and some thought it may cause nervousness among patients due to the topic area.

**Conclusions:**

A decision aid for shared advance care planning for severe COPD was found acceptable to most COPD patients and their doctors. However, many patient participants did not demonstrate understanding of the treatment options or prognostic estimates. Many participants endorsed the use of the decision aid between doctors and their patients with COPD, although they desired more information about quality of life. The design must optimize comprehensibility, including revising the presentation of statistical information in the icon array, and feasibility of integration into clinical workflow, including shortening the decision aid.

## Introduction

Chronic obstructive pulmonary disease (COPD) is a progressive disease affecting approximately 6.3% of adults (15 million) in the United States [1] and is the third leading cause of death in the United States [2]. As COPD advances, patients may experience COPD exacerbations—episodes in which their symptoms suddenly worsen, requiring hospitalization and a potential decision about whether to accept intubation. Patients in this situation who do not have advance directives often receive default invasive treatments, such as mechanical ventilation, which may not align with their goals of care [3]. Advance care planning (ACP) includes establishing advance directives and often involves discussions between the patient, family members, and outpatient clinicians [4]. Although most patients are open to discussing end-of-life issues, few have had such conversations with a doctor [5,6].

One strategy that has been advocated for improving patient-clinician communication is shared decision making [7]. Shared decision making is a process during which the clinician and patient work together to arrive at a decision that takes into consideration the patient’s preferences. Decision aids are tools that can encourage informed, shared decision making by providing information to patients regarding their condition, available treatment options, and potential outcomes, and can also help them identify and communicate their preferences [8].

Decision aid experts have developed standards for the creation of high-quality decision aids [9], including following a systematic development process and performing iterative usability testing with patients and clinicians [10]. Usability testing is conducted with intended end users completing specific tasks using the decision aid prototype, while performance data are electronically captured and/or an observer records notes on what they do or say [11]. The purpose of usability testing is to identify specific problems that prevent users from reaching the goals of the decision aid—in this case, to be able to participate in shared decision making about advance directives on whether to receive invasive mechanical ventilation. Recommended solutions to usability problems are then incorporated into the decision aid during the iterative design process.

We have developed a Web-based decision aid called InformedTogether, which is designed to support shared advance care planning between severe COPD patients and their doctors. In this manuscript, we outline the results of usability testing of InformedTogether. Other decision aids about advance directives have been developed for COPD patients [12,13]. InformedTogether differs from these decision aids because it is intended to be used by the doctor and patient together during the clinic visit (ie, shared decision making) and then be made available to either party to access individually online. InformedTogether also includes personalized prognostic estimates using a published decision model based on the best available evidence of COPD outcomes [14,15]. Providing doctors with prognostic estimates may facilitate advance care planning [16] because uncertainty around a patient’s illness trajectory has been identified as one reason doctors are reluctant to discuss end-of-life care planning [17,18]. The objective of this paper is to describe the usability of InformedTogether in terms of whether patients and doctors could use it to engage in tasks required for shared decision making about advance directives, whether they thought it was acceptable, and how the decision aid could be improved.

## Methods

### Development of the Decision Aid Prototype

The development and initial testing of InformedTogether was guided by the International Patient Decision Aid Standards (IPDAS) Collaboration criteria for quality decision aids [19]. The decision aid was designed to incorporate principles of shared decision making, including presenting patients with information about their treatment options and likely outcomes, presenting the risks and benefits of each option, and engaging the patient and physician in a conversation about the patient’s preferences [7]. The decision presented is which advance directive to choose in the event of acute respiratory failure: (1) Full Code, which allows intubation for mechanical ventilation, or (2) Do Not Resuscitate (DNR), which does not allow invasive mechanical ventilation, but permits noninvasive ventilation with bilevel positive airway pressure (BiPAP) or continuous positive airway pressure (CPAP). InformedTogether was designed to be used on a Web-based platform, either on a tablet computer or on a desktop. Our previous research indicated that patients and doctors would be comfortable using the computer together during the clinic visit, and that patients would be open to their doctor using a decision aid [20].

### Content of the Decision Aid Prototype

The decision aid allows clinicians to enter patient information including name, gender, and age. It then displays projected survival outcomes based on patient age and disease severity. The version used for this study calculated estimated outcomes for a hypothetical patient aged 65 with severe COPD. Pages include a description of the goals of the decision aid, personalized survival estimates for Full Code versus DNR advance directives based on patients’ age and severity of COPD, and suggested scripts for discussing the topics of prognosis and planning in case of a COPD exacerbation (see Multimedia Appendix 1 for decision aid screenshots).

### Expert Consultation

Once the prototype was developed, we solicited feedback from experts not involved in the development and usability testing of the decision aid. We consulted experts in human factors engineering, health risk communication, and health care decision making to get feedback on interface design, and consulted palliative care experts on content and wording. We also consulted a patient advocate to provide feedback on content.

### Usability Testing

#### Overview

**Figure 1:**
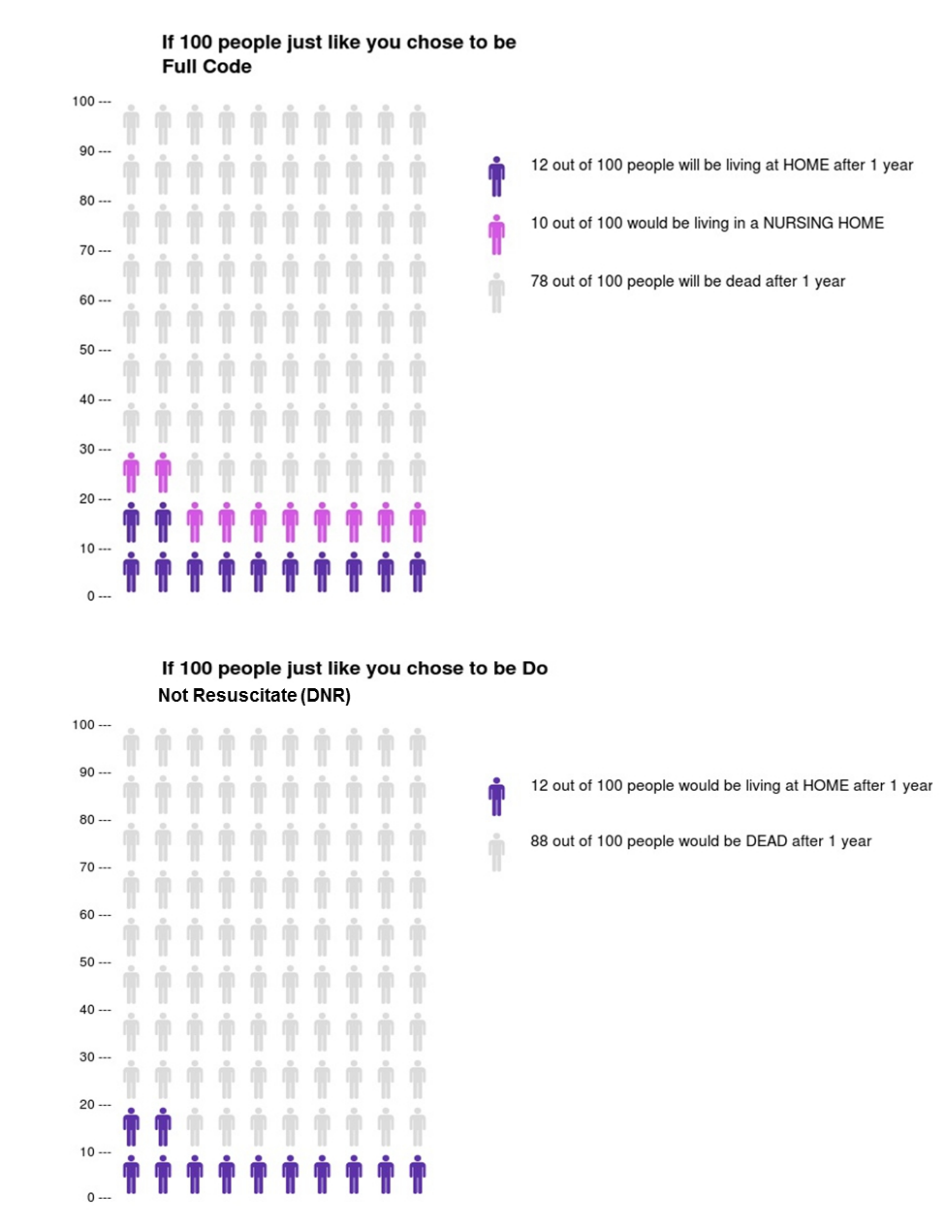
Icon arrays presented in phase 1 of usability testing. Likely outcomes 12 months after hospitalization for acute COPD exacerbation are shown for 100 hypothetical patients choosing either a Full Code or DNR advance directive.

Usability testing was conducted in two phases. The first phase focused on the icon array risk communication page, and the second phase tested interactions with the entire decision aid. An icon array—sometimes called a pictograph—is a graphical display of a number (usually 100 or 1000) of stick figures, circles, or other icons which represent individuals at risk of an event. The icons are shaded in one color to depict that they were affected by the event and unshaded to depict that they were not affected (see Figure 1). The icon array was created by a program developed at the Risk Science Center and Center for Bioethics and Social Sciences in Medicine, University of Michigan [21]. Usability testing focused on communication and understanding of treatment options, risks and benefits, and likely outcomes. Communication and understanding of patients’ values and preferences, an important component of shared decision making, was not assessed as this feature was not included in the decision aid at the time of the studies. We also explored whether the decision aid would be feasible to implement in a real-world clinic setting by asking questions about acceptability.

#### Phase 1: Patient Usability Testing of Icon Arrays in the Decision Aid

Phase 1 of usability testing was conducted over 1 week at the outpatient pulmonary clinic at Bellevue Hospital Center, a public hospital in New York City. The study protocol was approved by the NYU School of Medicine Institutional Review Board and by Bellevue Hospital Research Administration. Adult (18 years of age or older) English- or Spanish-speaking patients were approached in the waiting room and invited to participate. Interviews had two parts. The first part was designed to assess patients’ and doctors’ attitudes, knowledge, and preferences toward both shared decision making in general and shared end-of-life decision making. Those results are presented elsewhere [22]. The second part of the interview asked patient participants to view printed versions of the icon arrays (see Figure 1), and to explain what the pictures were showing and the meaning of Full Code and DNR in their own words. Participants were then asked about acceptability in terms of whether they would want their doctor to show them the icon arrays and whether they thought the icon array would help them make end-of-life plans or decisions. They were also asked about suggestions for improving the decision aid [23]. Interviews were audiotaped and transcribed.

#### Phase 2: Patient and Doctor Usability Testing of the Entire Decision Aid

Phase 2 of testing was conducted at a different center, with pulmonary rehabilitation patients and pulmonary doctors at Long Island Jewish Medical Center in New Hyde Park, NY. The protocol was approved by the NYU School of Medicine and the North Shore-Long Island Jewish Health System Institutional Review Boards. Adult (18 years of age or older) English-speaking patients with advanced-stage COPD who were receiving pulmonary rehabilitation were eligible for the study. All pulmonary doctors present during the day of the study were eligible. Each participant was shown the decision aid while being observed by a researcher. With patient participants, the researcher acted as the “doctor” and went through the decision aid on a tablet computer. Doctor participants used the decision aid on a laptop computer running Hypercam screen capture software (Hyperionics Technology LLC) and were asked to use the decision aid as if they were with a patient. Tasks analyzed included the following: (1) a click-through task, where users clicked through the decision aid, looking at the decision support materials while thinking aloud (ie, verbalizing their thoughts), (2) a graph interpretation task, where they were asked to respond to, and interpret, a graph, and (3) an icon array interpretation task, where they were asked to respond to, and interpret, an icon array. We then administered a brief, semistructured interview to all participants to assess their knowledge and understanding of the treatment choices presented in the decision aid, the acceptability of the decision aid with regard to the length, clarity, and amount of information, whether the participant would recommend use of the decision aid, and whether they had suggestions for improvements. Examples of questions to assess knowledge and understanding included “What did you think the overall message of the decision aid was?” and “In your own words, what is meant by ‘Full Code’?” Questions to assess acceptability followed guidelines established by the Patient Decision Aids Research Group, [23] and included “How would you rate the amount of information in the decision aid?” and “Did the decision aid make you feel nervous or fearful?”

### Data Analysis Methods

#### Phase 1: Patient Usability Testing of Icon Arrays in the Decision Aid

We performed a thematic analysis of the transcribed audio recordings as recommended by Boyatzis [24]. The analytical process involved the following: (1) generating codes to be attached to similar quotes or topics across transcripts, (2) comparing and contrasting ideas related to the codes to create themes that fit the nature of the data, and (3) assessing the reliability of codes and themes. Data were analyzed keeping in mind key usability measures, such as understanding of the treatment choices (Full Code and DNR) and acceptability of the icon arrays. Qualitative data were coded and analyzed using NVivo qualitative data analysis software, version 10 (QSR International Pty Ltd).

#### Phase 2: Patient and Doctor Usability Testing of the Entire Decision Aid

For analysis of the click-through and think-aloud tasks, transcribed audio recordings of participant interactions with the decision aid were time-stamped (ie, video time codes were added into the file) and annotated for interesting user interactions and comments and to identify usability problems. The codes from the analysis were then summarized in terms of type and potential impact. For analysis of the interviews, closed-ended questions were summarized with descriptive statistics, and answers to open-ended questions were analyzed thematically as described above.

## Results

### Phase 1: Patient Usability Testing of Icon Arrays in the Decision Aid

#### Sample

Out of 52 eligible patients, 11 consented to participate in the study. The most common reason given for declining to participate was lack of time. Patient participants were mostly male (6/11, 55%), Hispanic or Black (10/11, 91%), and had a median age of 60 years, ranging from 23 to 73 (see Table 1). Most participants (7/11, 64%) had a high school education or less.

#### Understanding of Treatment Choices and Likely Outcomes

Participants understood that the chance of survival at 1 year was better with Full Code, and that survival was low regardless of advance directive. One participant said the following:

There’s a better chance with the Full Code than the no resuscitation. The Full Code is with the tube right? It shows them that they could be longer, you know, you could help them out more with the Full Code. With the DNR you ain’t got no chance. They ain’t got no chance out of 100. (With Full Code) they got little chance out of 100.

Male, 49, black/African American

Some patients identified Full Code as *better* in terms of survival but had difficulty understanding the potential trade-off that more survivors of Full Code would be institutionalized within a nursing home. For instance, one participant started out saying “I would choose this one (Full Code) because then there would be a chance for longer life.” After the interviewer pointed out that almost half of those who survive would be living in a nursing home, the participant said the following:

In that case, I wouldn’t like that because I had to make a decision like that with my father and I decided to keep my father at my house and treat him at home. I would choose to live at home because I don’t agree and have never agreed with being in a nursing home because the people who are in a nursing home die faster.

Male, 60, Hispanic

There was variable understanding of the meaning of the two advance directives. Although many participants responded in terms of whether the patient would get “the tube”, many responded by describing survival outcomes for that advance directive. These answers did not describe what the advance directive meant in terms of treatments allowed. For example, one patient participant (male, 51, Hispanic) responded that DNR meant “...that they don’t have life, that they die.” Another participant (female, 35, Asian/Asian American) responded that Full Code meant “...you live longer...this is group of people, you’re more statistically will prolong your life.” Furthermore, several patient participants did not understand that choosing DNR could still mean patients could be treated with a breathing mask noninvasively.

Several participants misinterpreted the icon array in various ways. For instance, one participant thought that the numbers on the vertical axis represented age. Another considered *code* as referring to cardiac arrest and DNR to pertain only to the heart. When asked if patients who are DNR could get the breathing mask, the participant said the following:

They can get intubated as well because my concept of it is your heart has to stop or brain damage, things like that, but if your brain’s dead, your brain’s dead. If your heart stops that’s when a resuscitation, but if you just coming in because you can’t breathe I don’t think that falls under the...As long as the heart is pumping, treatment can be given, but if that heart stops treatment cannot be given.

Male, 34, black/African American

**Table 1:** Participant characteristics.

Characteristics			Patients		Doctors
			Phase 1 (n=11)	Phase 2 (n=4)	Phase 2 (n=8)
**Demographics**					
	** Gender ^a^, n (%)**				
		Female	5 (45)	1 (25)	N/A
		Male	6 (55)	3 (75)	N/A
	**Race/ethnicity, n (%)**				
		Hispanic/Latino	7 (64)	0 (0)	0 (0)
		Black/African American	3 (27)	2 (50)	0 (0)
		White	0 (0)	2 (50)	3 (38)
		Asian/Asian American	1 (9)	0 (0)	4 (50)
	Age in years, median (range)		60 (23-73)	72 (57-76)	33 (28-43)
	**Highest education level completed, n (%)**				
		8^th^ grade or less	2 (18)	0 (0)	N/A
		9^th^ to 12^th^ grade	5 (45)	0 (0)	N/A
		Some college	2 (18)	0 (0)	N/A
		College degree	2 (18)	4 (100)	N/A
	Years of training after residency, median (range)		N/A	N/A	2.5 (1-5)
**Clinical characteristics**					
	**Self-rated general health, n (%)**				
		Excellent	1 (9)	0 (0)	N/A
		Very good	2 (18)	0 (0)	N/A
		Good	2 (18)	2 (50)	N/A
		Fair	4 (36)	1 (25)	N/A
		Poor	1 (9)	1 (25)	N/A

^a^Data on gender were not collected for doctors.

#### Acceptability

Overall, patient participants endorsed the use of the decision aid between patients and doctors. Participants articulated that death is “reality” and that patients need to know their options. Some patients said the decision aid should be used with family members present, and a few raised the concern that seeing the figures either caused them to be fearful or may cause fear in other patients. About one-third of patient participants said that the icon array would be helpful for patients in planning for end-of-life and about half said that they would like their doctors to show them a picture like the icon array (see Table 2).

**Table 2:** Results of patient interviews from phase 1 of usability testing.

Questions		Responses (n=11), n (%)
“**Would these pictures be helpful for patients to see?”, n (%)**		
	Yes	4 (36)
	No	1 (9)
	Missing	6 (55)
“**Would you like your doctor to show you something like this?”, n (%)**		
	Yes	5 (45)
	No	1 (9)
	Missing	5 (45)
“**Do you think this would help you make plans or decisions about what you would want to happen at the end of your life?”, n (%)**		
	Yes	4 (36)
	No	1 (9)
	Missing	6 (55)

#### Suggestions for Improvements

When asked for suggestions for improving the decision aid, patient participants suggested increasing the size of the images and font to enable people to see them more clearly. Participants also wanted more information about outcomes such as the chance of successfully weaning from the ventilator. As one patient (male, 34, black/African American) stated, “It (the decision aid) should be something that forewarns you as far as what happens if you get intubated, as far as, like, there’s a twenty percent chance it might never come out.” A few participants who couldn’t define DNR or Full Code requested the definitions to be included right next to the icon array images. For example, one participant stated the following:

I don’t understand, like, what’s really going on in the pictures and where the numbers come in, but yeah, I need some more information. This sheet, it’s just saying Full Code and DNR, and also explain what Full Code means because...I think you know anyone that’s not knowledgeable about these terminologies, it’d be good to break it down in simpler terms and explain exactly what these images are representing here.

Male, 23, black/African American

### Phase 2: Patient and Doctor Usability Testing of the Entire Decision Aid

#### Sample

Four out of 5 (80%) patients and 8 out of 8 (100%) doctors that were approached consented to participate in the study. Table 1 provides demographic and clinical characteristics of the participants. Patient participants had a median age of 72 years (range 57 to 76), and rated their COPD as severe or very severe. All participants except for 1 (3/4, 75%) reported having an advance directive, but only 1 (1/4, 25%) participant reported having had an end-of-life discussion with their doctor. Doctor participants had a median age of 33 years (range 28 to 43), and all (8/8, 100%) reported having end-of-life discussions with their patients.

#### Understanding of Treatment Choices and Likely Outcomes

##### Patient Participants

Several patients responded to questions about what was meant by the terms Full Code and DNR by expressing their values and thoughts about that directive instead of by giving an objective definition or description of the term (see Table 3). For instance, one participant (male, 76, white) described DNR as the following: “If you have no chance of recovering, don’t do all these things and you die soon anyway.” Often, the patients did not express the level of understanding that was expected after using the decision aid. For example, one patient (male, 68, black/African American) defined Full Code as meaning “Let your wishes be known ahead of time.” Change in knowledge could not be determined because there was no baseline assessment before viewing the decision aid.

When asked to interpret the survival curve and icon array, 2 out of 4 (50%) patients either interpreted some aspect of the icon arrays incorrectly or stated that they thought the icon arrays needed more clarity. Of note, 1 (1/2, 50%) of the patients (male, 57, black/African American), who later interpreted aspects of the icon array incorrectly, stated that he thought the decision aid was “...easy to see and understand,” highlighting the necessity of using specific measures to assess understanding.

**Table 3:** Responses to questions assessing understanding of treatment options from phase 2 of usability testing.

Questions	Responses	Patient gender, age in years	Expresses understanding?, Yes/No
**In your own words, what is meant by** ***Full Code*** **?**			
	When you are using oxygen or CPAP or the hose. Make the best of the situation and live.	Male, 57	No
	Code is when you’re dying.	Male, 76	No
	Let your wishes be known ahead of time.	Male, 68	No
	CPR, meds, cardiac life support, intubate.	Female, 75	Yes
**What is meant by** ***DNR*** **?**			
	If you have no chance of recovering, don’t do all these things and you die soon anyway.	Male, 76	No
	Do not resuscitate.	Male, 68	No
	Do not resuscitate. Means don’t do this, just do palliative care, leave me alone, make me comfortable even if it means drugs that may hasten my death.	Female, 75	Yes
	Gamble that you will pull through. Do not revive me. I’m giving up.	Male, 57	Yes

##### Doctor Participants

When asked to describe the survival curve and icon array as if they were with a patient, 3 doctors out of 8 (38%) stumbled over some aspect of describing the icon array, including one who described the shaded icons as representing people who did not survive the initial hospitalization. However, the icon array depicted outcomes in the 12 months after hospitalization. Another doctor described the icon arrays as showing 100 people with severe COPD who were “...divided into two groups,” instead of as alternative treatment scenarios for the same people. Doctors’ thoughts on the comprehensibility of the survival curve and icon array for their patients were mixed. Two doctors liked the survival curve better but thought the icon array may be more understandable to patients. Two doctors cautioned that the survival curve may not give patients all the information they need to make a decision, since it only provided information on survival, and not quality of life. One (1/8, 13%) doctor suggested that both figures be removed from the decision aid. One doctor stated that regardless of the graph, the physician should guide the patient in reviewing and interpreting it, saying, “A lot of people use numbers to guide their decision process. But (numbers) should be interpreted with caution. It’s important to guide the patient in reviewing the graphs.” Yet another doctor suggested including scripts to aid the doctor in describing the figures to patients.

#### Acceptability

##### Patient Participants

All patient participants (4/4, 100%) expressed interest in having their doctor use InformedTogether with them and stated that they would recommend their doctor use the decision aid with other COPD patients, suggesting demand for use of the decision aid. Patients recommended use of the decision aid in order to help provide information and options, and to achieve better decisions. For example, one patient (male, 76, white) said he would recommend that his doctor use the decision aid “...with all patients. Doctors should give patients the truth. The more information patients have, the better decision they can make.” Most (3/4, 75%) patients reported not feeling nervous or fearful after using the decision aid. One patient (male, 57, black/African American) acknowledged that talking about death caused him “a little” nervousness or fearfulness, but still concluded that “...you’ve got to (have such discussions). The sooner the better. Don’t beat around the bush.”

##### Doctor Participants

All doctors (8/8, 100%) responded that they would recommend doctors use the decision aid with their patients. The most commonly cited reason was that it would help facilitate important discussions around end-of-life treatment options (4/8). One doctor (30, white) pointed out it would also facilitate earlier discussions, saying, “(We) need to have this conversation, but doctors are short on time. These conversations happen in the hospital, which is the wrong time.” Out of 8 doctors, 3 (38%) mentioned concerns about time constraints during the clinic visit with regard to whether they would suggest use of the decision aid and its practicality for use within a regular clinic visit. For instance, one doctor (30, white) said the decision aid was “...probably too long for a regular clinic visit but the length is appropriate for this type of discussion.” The time needed to use the decision aid ranged from approximately 15 to 20 minutes (10 to 15 minutes for the pages to be shared with the patient).

#### Suggestions for Improvement

##### Patient Participants

Patient participants expressed their desire for more information about treatment options such as lung transplant and BiPAP/CPAP, and for information about the quality-of-life implications of the different treatment options. For instance, patients wanted to know whether mechanical ventilation would be permanent and what would happen in the future with the choices presented.

##### Doctor Participants

Doctors also agreed with patient participants that the decision aid should provide more information about the implications of the choices, including quality of life and functional status. Some doctors (3/8, 38%) mentioned the topic area as a reason for potential nervousness among their patients and several (4/8, 50%) said that the decision aid may make patients feel nervous or fearful, depending on the patient. One doctor (28, Asian/Asian American) said, “Some (patients) don’t like to discuss advance directives. It causes anxiety, but it depends on the patient.”

Analysis of the think-aloud and screen capture data collected yielded a number of other potential usability issues, which can be subsumed into content, consistency, layout, orientation, links, and feedback issues. For example, a layout problem experienced by several doctors was that they did not readily recognize that there was more information, such as the *next* and *back* buttons, below the visible screen and that they had to scroll to see all the information on the page. Usability issues and corresponding recommended changes to the decision aid are described in Table 4. Results are organized into short-term changes, which may be implemented easily without further research, and long-term changes, which require further research to understand how best to implement.

**Table 4:** Usability issues arising during phase 2 of usability testing and recommended decision aid changes.

Areas		Usability issues	Participants, age in years	Recommended changes
**Short-term changes**				
	**Content**			
		Lack of axes labels for figures.	Doctor, 43	Add axes to figure labels.
		Unclear which screens are meant to be shared with the patient.	Doctors, no age given, 28, 33	Add a note to doctors on the page before the start of screens meant to be shared with patients alerting them to share upcoming screens. Provide proper training/orientation prior to use.
		Pictures of patients depicting intubation do not show the tube clearly.	Patient, 75	Change the pictures used to show intubation.
		A page with definitions of advance directives was redundant.	Doctors, no age given, 31	Remove page with redundant definitions.
	**Consistency**			
		The figure for patients was described as “people out of one hundred“, but the axis scale was written as proportions of 1.		Change axis to match wording.
		*Back* and *next* buttons are not located consistently but move depending on how much information is on the page.	Doctor, 33	Make location of buttons consistent across each page.
	**Layout**			
		Had to scroll to see all information—some users had difficultly realizing more information was below visible screen, such as the *back* and *next* buttons.	Doctors, 28, 33, 33, 33	Make graphic smaller to eliminate need for scrolling, reducing burden on working memory.
	**Orientation**			
		Some confusion, and took some time for doctors to get oriented to survival curve.	Doctors, no age given, 31	Add a suggested script for doctors to use when describing curve to patients. Provide proper training prior to use
		Unsure about what *Resources* link would lead to.	Doctor, 33	Make the name more descriptive.
	**Links**			
		Broken links on *Patient Resources* page led to webpage without information on advance directives.	Doctor, 33	Update links.
		Links should open in a new tab/window instead of replacing the decision aid in the window.	Doctor, 33	Reprogram so that links open in a new tab or window.
	**Feedback/ links**			
		Links on *Resources* page should change color once visited.	Doctor, 33	Change link color once it has been clicked on/visited.
	**Feedback**			
		Users unsure of how to finish using the decision aid and how to close it.	Doctor, 33	Change *I’m done* button to say *Exit*—put it in a more visible area.
		Unclear that last page is last page of decision aid.	Doctors, 33, 43	Add text box that appears after clicking to exit, making it clear the user has reached the end of the decision aid.
**Long-term changes**				
	**Content**			
		Icon arrays need more clarity.	Patient, 75	Scripts. Further usability testing and refinement.
		Lack of information regarding quality of life/functional status for patients in nursing homes, with intubation, and with BiPAP.	Doctors, 33, 30, 31, 43	Add more information about quality of life with various treatments and places of care.
		Probably too long for a regular clinic visit but the length is appropriate for this type of discussion.	Doctor, 30	Find potential areas to cut. Discussions with doctors about implementation.

## Discussion

### Principal Findings

Some patient and most doctor participants were able to use the decision aid to complete tasks required for shared decision making, although many patients had difficulty articulating the treatment options and understanding the icon arrays used to communicate estimated prognoses for each option. Many patient and doctor participants rated InformedTogether highly on measures of acceptability, including endorsing the use of the decision aid between doctors and their patients with severe COPD.

Usability testing provided insights into modifications that could improve usability of the decision aid, including minor issues related to content, layout, links, and feedback to the user. Several problems with comprehension were uncovered, especially with regard to understanding the icon array and the meanings of the two advance directives. Patient and doctor participants also suggested content to add, including quality-of-life implications of the advance directives. These content suggestions reflect important information needs to enable patients to engage in informed decision making using the decision aid.

### Implications for Decision Aid Redesign

Next steps to continue development of InformedTogether will include adding information on quality of life and functional status when patients are intubated and when they are discharged to a nursing home. In addition, further testing of the revised icon array is needed to ensure the decision aid will be understandable to the majority of patients. Although participants in these phases of usability testing struggled to interpret the icon array correctly, other research has found that icon arrays are readily understandable to patients. In studies of alternative ways of presenting risk estimates in decision aids, icon arrays have been shown to be understandable to a majority of users and result in higher comprehension levels compared to other methods of displaying information [25-27]. Potential explanations for our results include poor labeling of the icon arrays, or differences in the demographic characteristics of the patient population. Another explanation could be low graphical literacy among our sample, which was not formally measured. Visual aids such as icon arrays have been shown to be especially helpful for communicating probabilities for people with low numeracy but with relatively high graphical literacy [28,29]. It may be that our population had low numeracy and low graphical literacy and, thus, icon arrays were not helpful in aiding comprehension of the statistical data. Other studies of icon arrays have found that up to 70% of individuals with low numeracy still answered risk understanding questions incorrectly, depending on their graphical literacy [29,30]. One study found that individuals with low graphical literacy had better comprehension when shown numbers instead of graphs [28]. Subsequent usability testing of InformedTogether will involve formal measures of participants’ numeracy and graphical literacy [31]. We will also explore the possibility of having alternative presentations of risk data available, which can be tailored to participants’ numerical or graphical literacy, or can be chosen by patient users themselves. IPDAS standards recommend allowing the patient to choose how they view probabilities—either in numbers, words, or figures [19]. To optimize patient understanding and usefulness of the decision aid to patients with low numeracy and graphical literacy, it may be important to train health care providers on how to best communicate the risk information in the decision aid to ensure that it is understood by patients with different literacy levels [32]. Once revisions have been made to the icon array content based on the results of these phases of testing, we will again evaluate understanding and compare comprehension of the icon array with that of alternative forms of risk data presentation.

We will also need to improve the communication of information about the different advance directives and the questions used to test comprehension. Several patients offered their opinion or feelings about each directive rather than giving a definition in terms of allowable treatments. Less equivocal results on patients’ understanding of advance directives may have been achieved by using closed-ended questions instead of, or in addition to, open-ended questions. For example, participants could be asked “Does a patient choosing a Full Code directive wish to allow intubation?” Future testing will include closed-ended questions as well as a baseline assessment of knowledge.

Revisions must also focus on allowing the decision aid to fit within the workflow in terms of time constraints. In addition, the decision aid must also support doctors’ attempts to establish rapport with their patients. In future testing, we will specifically elicit users’ attitudes about the effect of the decision aid on doctor-patient rapport. Finally, one component of shared decision making that had not yet been developed in this version of InformedTogether was patient preference elicitation and the communication and comprehension of these preferences. We recognize that including methods for values clarification and communication is an IPDAS criterion and an essential component of patient decision aids [19]. However, we intended to first test whether patients understood the information in this prototype before adding values clarification. Future versions will incorporate this central feature of shared decision making and test this feature for usability. Other relevant IPDAS criteria that were not met due to the early stage of the prototype include providing structured guidance for deliberation and discussion of the decision with others, complete information on the evidence used in the decision aid, including references and the quality of scientific evidence, and information about the developers and their conflicts of interest.

### Results in the Context of Other Studies of COPD Decision Aids

Other investigators have tested the communication of statistical information to COPD patients in usability testing of decision aids related to mechanical ventilation and other aspects of COPD treatment. In the evaluation of their decision aid for COPD, Wilson et al reported that prior to the evaluation study, their decision aid had undergone several revisions, although they did not describe iterative usability testing in detail [33]. Their audio booklet decision aid for mechanical ventilation was tested by severe COPD patients, about half of whom had a high school education or higher. Similar to the present findings, the majority of users (88%) reported that the decision aid was “not at all” difficult to understand, however, in analysis of open-ended questions to ascertain understanding, almost one-quarter of participants showed an inadequate comprehension of the options presented in the decision aid [33]. These results highlight the usefulness of a mixed-methods approach for a deeper understanding of results. In our studies, only 3 patients out of 15 (20%) reported that the decision aid made him or her feel nervous or fearful, but in Wilson et al’s study, almost half the participants (15/33, 45%) found the decision aid to be at least a little upsetting. These results underline that it is important for doctors to assess their patients’ readiness for using the decision aid in order to address any nervousness or discomfort.

For their computer-based decision aid regarding inhaled steroid therapy for COPD, Akl et al described usability testing consisting of interviews with 7 COPD patients followed by pilot-testing with 8 COPD patients [34]. Similar to the findings from our studies, the main modifications based on usability testing included changing the presentation of statistical information and the amount of information. Results from their pilot test showed improvement in knowledge from baseline, but users did not rate the clarity of statistical information highly [34]. The best ways of presenting statistical information to patients, including risk information, is an ongoing challenge that deserves further study.

### Limitations and Strengths

As described above, one limitation of our study was the way comprehension of treatment options was measured. Inclusion of closed-ended knowledge questions, as well as a baseline measure of knowledge, would have helped clarify what effect, if any, the decision aid had on understanding of treatment options. A further potential limitation was that doctor participants may not have been given adequate time to orient themselves to the decision aid before testing. Although they were provided with 5 minutes to look through the pages before recording began, the decision aid is meant to be used by doctors who have had formal training with the tool and the results of our testing may, therefore, not fully reflect the intended use. For example, doctors’ errors in interpretation of the icon arrays may have been avoided if they had been given the orientation and training in use of the decision aid that is planned for when the tool is disseminated.

We did not include testing of the decision aid during a clinic consultation for these early rounds of usability testing. This paper reports initial usability testing of a decision aid prototype which is being developed further before conducting feasibility testing in the clinical setting. Feasibility testing is planned to measure whether the decision aid promotes shared decision making and informs patients, and will measure outcomes such as demand and implementation, as well as patients’ preparation for decision making, motivation to make advance care plans, confidence in decisions, and patient-doctor communication. Feasibility testing will also measure knowledge, comprehension, and acceptability. It is unknown whether testing within the clinic would show similar levels of comprehension or acceptability, however, our study was designed to enable critical revisions to the prototype before testing in the clinical setting.

Patient participants in phase 2 of usability testing were well educated (ie, all had college degrees) and their responses may not represent those of patients with broad educational backgrounds. However, patient participants in phase 1 had a larger range of education attainment, with most having completed 12^th^ grade or less. Another limitation related to our sample was the small sample size, however, experts recommend 6 to 12 participants to detect the majority of usability problems [35]. While small sample sizes are adequate to uncover most usability problems, the small sample size may have affected the results regarding acceptability. For example, patients who agreed to participate in the study may have been more open to discussing end-of-life issues, and thus more likely to recommend use of a decision aid about end-of-life decisions. In phase 1, most eligible patients declined to participate. Although we did not systematically gather data about decliners, the reason most often given for declining was lack of time. We recruited patients from the waiting room of a clinic where it is common for patients to experience wait times of 2 hours or more, and asked them to stay for approximately 30 minutes after their appointment to participate. Those who participated may have been less likely to have commitments, such as full-time jobs or child or eldercare responsibilities, that would have prevented them from being able to stay longer at the clinic. The younger average age of the sample of doctor participants may have influenced the results if, for example, younger doctors tend to be more comfortable using computers or less worried about the impact of a computer-based tool on the patient-doctor relationship. We did not, however, measure doctors’ comfort with, or preferences for, using computer-based tools with their patients. Subsequent usability testing, which is ongoing, involves doctors with varying levels of seniority. We also did not test the effectiveness of the decision aid in terms of constructs related to the decision-making process and decision quality, such as preparation for decision making [36].

Our study has several strengths, including the formal evaluation of screen capture recordings to identify usability problems. In addition, our sample included intended end users—doctors who treat COPD patients and COPD patients from two different clinic locations. Furthermore, we included patients from racially and socioeconomically diverse backgrounds. Our results provide directions for further refinement and development of the decision aid to ensure that it is usable and useful to both patients and doctors.

## Appendix

Multimedia Appendix 1 Screenshots of the InformedTogether decision aid.

## Abbreviations

ACP: advance care planning
BiPAP: bilevel positive airway pressure
COPD: chronic obstructive pulmonary disease
CPAP: continuous positive airway pressure
DNR: Do Not Resuscitate
IPDAS: International Patient Decision Aid Standards
IRTI: Interdisciplinary Research Training Institute
NIDA: National Institute on Drug Abuse
