# Supplementary material for: InformedTogether: Usability Evaluation of a Web-Based Decision Aid to Facilitate Shared Advance Care Planning for Severe Chronic Obstructive Pulmonary Disease
Source: JMIR Hum Factors. 2015 Feb 25;2(1):e2. doi: 10.2196/humanfactors.3842 (PMC4797670; doi:10.2196/humanfactors.3842)
Supplement: Multimedia Appendix 1 [file humanfactors_v2i1e2_app1.pdf]

COPD - Decision Aid

copd-da.herokuapp.com/pages/overview

NYU COPD Decision Aid | Add a New Patient | Existing Patient | Schedule | clinician | Logout

Page 1 | I'm Done

# Overview

This decision aid is about **outcomes for patients with severe COPD** and includes language that you can use to talk about these outcomes with your patients as well as age-specific estimates and resources for them to access from home.

First, **lets review some general estimates of outcomes** (based on clinical studies) for patients with severe COPD:

**4 year survival**
On average, patients have a **less than 60**% chance of being alive in 4 years time.

**1 year hospitalization**
On average, patients have a **greater than 50**% chance of being hospitalized for an acute COPD exacerbation within the next year.

> **6 month survival**
> Of patients who are hospitalized and admitted to the ICU with respiratory failure due to COPD, on average, patients have a **62**% six month survival.


*References:*
*Celli et al, NEJM 2004; 3 50:1005-12*
*Fitzgerald et al. Can Respir J 2007;14(3):145-152*
*Wildman et al. Thorax 2009; 64:128-32*


Next

Page 2 ✖ I'm Done

## Goals

# Of Decision Aid

Do you know if Jim has started to plan for when they become severely/ potentially terminal ill (sometimes known as "the end of life")?

What if Jim were admitted to the hospital tomorrow for a severe acute COPD exacerbation?

The **goal** of this decision aid is to **facilitate advance care planning -** to help patients plan for when they become severely/ potentially terminal ill (sometimes known as "the end of life").

It will provide you and your patients with **estimates** of **outcomes** with **different advance directives** specifically for mechanical ventilation for COPD exacerbation.

These estimates are individualized based on each patient's age.

Click on the "next" button to see estimated outcomes if Jim were admitted to the hospital tomorrow.

Back Next

COPD - Decision Aid

copd-da.herokuapp.com/pages/survival-comparison-full-code-vs-dnr

NYU COPD Decision Aid | Add a New Patient | Existing Patient | Schedule | clinician | Logout

Page | I'm Done

# Survival Comparison Full Code Vs. Dnr

**What if** Jim **were admitted to the hospital tomorrow for a severe acute COPD exacerbation?**

Jim's estimated **survival over the following 12 months** if she/he was admitted to the hospital for acute COPD exacerbation is shown below.

The survival curves show estimated survival for **Full Code** versus **Do Not Resuscitate (DNR)** advance directives.

**This graph is tailored to Jim's COPD severity and age.**

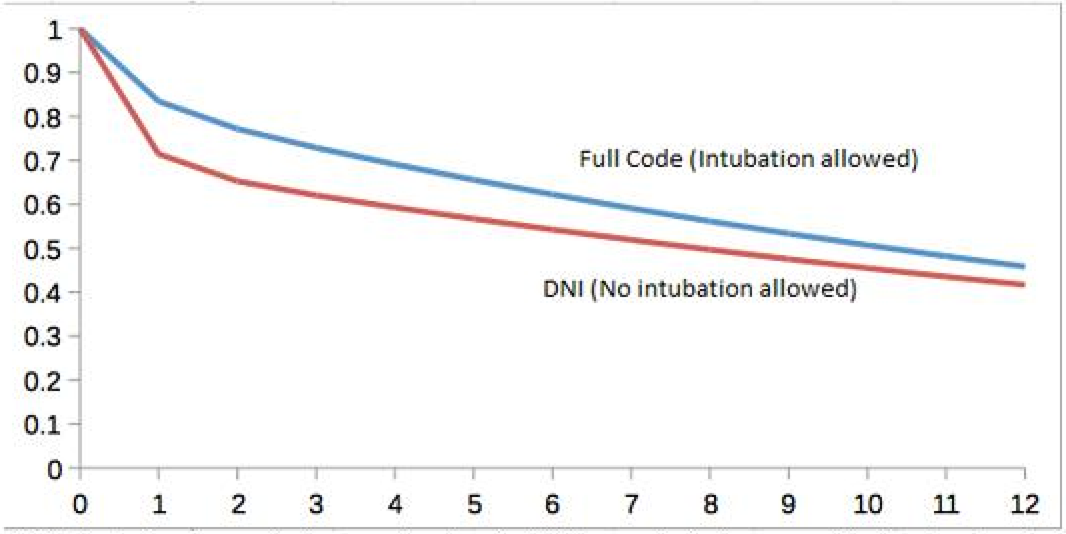


***Full Code** is a type of advance directive that tells people that you would allow treatment with a breathing machine in case you become very sick and cannot breathe on your own.*

***Do Not Resuscitate (DNR)** is another type of advance directive that tells people that you would NOT allow a breathing machine but you would allow oxygen through a mask and other medications.*

Back | Next

# Would You Like Help Communicating These Estimates?

If you would like, this decision aid can help you communicate these estimates with Jim, and help you start to talk about advance care planning.

Click on the "next" button for screens you can share with Jim.

Back  Next

# Patient Communication Script

(to the clinician: here are some suggested scripts for how to begin this conversation. Of course, feel free to augment as per your clinical judgement and relationship with your patient)

Jim, I would like to take some time to talk to you about your COPD/emphysema.

As you know, you have severe COPD/emphysema, and this may require that you use oxygen to help you breathe. COPD/emphysema is a disease that unfortunately gets worse with time.

Have you given any thought to what your wishes would be if you were suddenly need to be hospitalized?

May I have your permission to discuss with you what may happen if you were to be hospitalized for difficulty breathing (a COPD exacerbation)?

If **yes**, click "**Next**" to proceed.

If **no** [patient will be taken to the last page which is the list of resources with a blurb saying "Thanks for considering talking about advance care planning. Here is a list of resources that you may find useful. If, on the next visit you choose to talk more about this we can always try to use this decision aid again"]

Back   Next

I'm Done

## Treatment Choices

Patients with advanced lung disease, such as COPD may become very ill and unable to breathe on their own. This is called a **severe COPD exacerbation** and usually requires patients to be admitted to the hospital.

In the hospital, there are three types of treatments that can be given to patients:

| BiPAP/CPAP | Intubation | Comfort Measures Only (Palliative Care) |
|---|---|---|
| 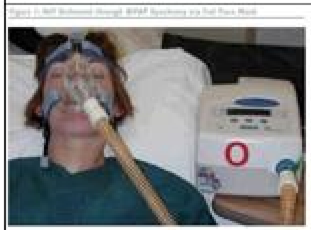 | 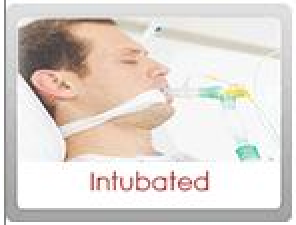  | 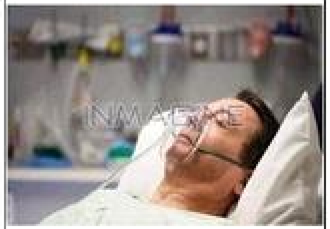 |
| **BiPAP/CPAP** is a breathing machine that uses a **mask** to blow pressurized air into the mouth and nose of the patient to help them breathe. | **Intubation** is a form of breathing machine that requires a **breathing tube** to be inserted into the mouth and into the lungs of the patient. | **Palliative care** is any form of medical care or treatment that reduces the severity of disease symptoms. This can be given in addition to other treatments such as a breathing machine, or just by itself |
| *Doctors usually try this first because there are less risks with this breathing machine* | *Doctors usually try this second, if BiPAP/CPAP does not work, because there are more risks with this breathing machine.* | *Doctors usually choose this type of treatment (comfort measures only), after discussion with the patient, if they believe they will not survive, or will have a poor quality of life with other treatments* |

In the next slides we will see some benefits and risks of these different types of treatments

Back

Next

# Benefits And Risks

**BiPAP/CPAP** has **fewer risks** when compared to **Intubation (the breathing tube)**. **But** BiPAP/CPAP **may not work** - at that point patients and their doctors need to decide if they will move on to intubation.

If they choose **NOT to continue on to intubation (Do Not Resuscitate/ DNR)**, patients will very likely **not survive** - this is the main reason why intubation is usually the next (automatic) step in treatment.

**The main benefit of Intubation therefore is that patients may survive longer - BUT you should know that intubation has risks:**

| Main benefits of INTUBATION | Risks of INTUBATION |
|---|---|
| | |
| • Breathing is easier because a machine is doing the breathing for the patient | • Discomfort with breathing tube |
| • Patient may **live longer** | • Not being able to talk |
| | • Not being able to eat food (although they will still get nutrients through a feeding tube or through their veins) |
| | • Usually not being able to walk |
| | • They may not be able to come off of the breathing machine and therefore may need to be placed in a long-term care home (nursing home) |

In either case, with a breathing tube or with a mask, doctors will do their best to make sure they make all patients as comfortable as possible - even if the treatments don't work and the patient is dying.

**The reason we are talking about this today** is so that - when the time comes – you will know about your options and can talk about this with their family. We would like to more sure you are **prepared** to make a **decision that feels right for you**.

With your permission, I can tell you about what might happen if you decide you would allow a breathing tube (Full Code); or a breathing machine and mask (DNR).

If yes, click **Next**

Back

Next

# Survival Comparison Full Code Vs. Dnr Patient View

OK, imagine there were 100 people like you - your age and with COPD.
If those 100 people had to go to the hospital because they couldn't breathe, this is what would happen within the **next 12 months**:

- If they chose to have a **Full Code advance directive** (to allow a breathing tube), about 54 out of 100 people would die.
- If they chose to have a **DNR advance directive** (to not allow a breathing tube but to allow a mask breathing machine called BiPAP/CPAP), about 58 out of 100 people would die.

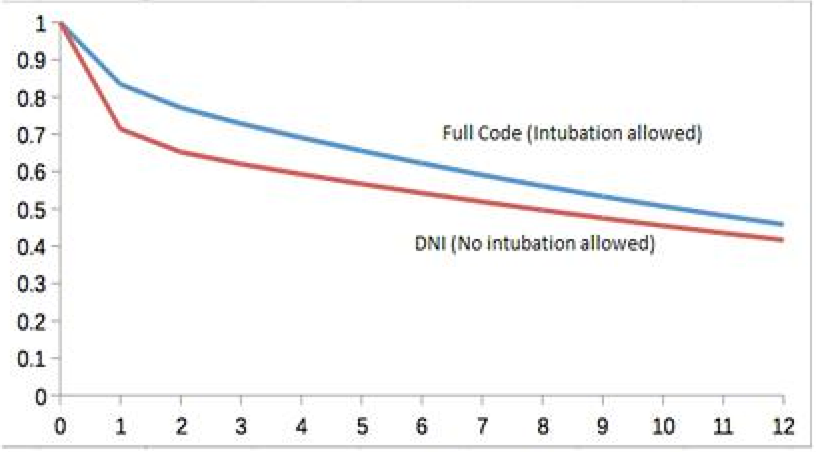


On the next screen we will see a picture of how many people who survive will be living back at home 12 months after they are hospitalized, and how many will need to be living in a **nursing home**.

A **nursing home** is a place where you may need to live if you become sick and unable to take care of yourself at home.

# What May Happen In The 12 Months After Hospitalization

Jim, if 100 people -who were your age and had your level of COPD were hospitalized tomorrow, and had to choose between whether they wanted to have  a breathing tube or not, these pictures show what would likely happen in the 12 months after they were hospitalized.

If patients decide to allow intubation this is called **FULL CODE**
If patients decide **not** to allow intubation this is called **DNR**

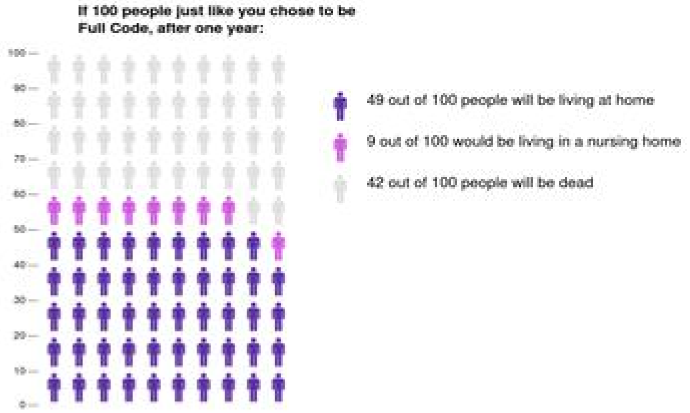


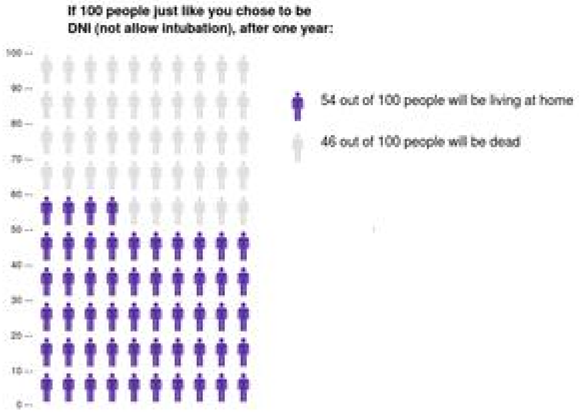

COPD - Decision Aid

copd-da.herokuapp.com/pages/advance-directives-definitions

NYU COPD Decision Aid | Add a New Patient | Existing Patient | Schedule | clinician | Logout

Page

I'm Done

# Advance Directives Definitions

Especially for people who have serious conditions like COPD, you can take time to decide that, if you need to be in the hospital, you **either** *want* **or** *don't want* a *breathing tube*. Deciding beforehand often means making an **Advance Directive**.

- If you *want a breathing tube*, you would have an advance directive called **Full Code**.
- If you *don't want a breathing tube*, you would have an advance directive called **Do Not Resuscitate**, or **DNR**.

**Advance directives** are decisions patients make before they become very sick so that their doctors and their families can know what they would decide if they become very sick and cannot speak for themselves.

**Full Code** is a type of advance directive that tells people that you would allow treatment with the **intubation** form of breathing machine in case you become very sick and cannot breathe on your own.

**Do Not Resuscitate (DNR)** is another type of advance directive that tells people that you would NOT allow a breathing machine but you would allow the BiPAP/CPAP mask breathing and other medications.

Back

Next

# The Start Of A Conversation

I know we have gone through a lot of information today and there will be a lot of questions on your mind. I would very much like for this to be **the start of a conversation between us and also with your family**.

Our goal today is **NOT to persuade** you to make a choice. Instead, the **goal is to make sure you are aware that you have a choice and what those choices are**. Also, I hope that this can invite you to talk with me and your family about your thoughts and wishes.

We will give you a username and password that you can use from home to look at this information with your family.

You will also see a list of several excellent resources for people who will be able to help you get more information and help you plan.
We should schedule another visit for you to come back and talk to us about your questions and plans if you decide to make any.
You could bring your family members with you at that time if you would like as well.

*Do you have any questions for me right now?*

COPD - Decision Aid
copd-da.herokuapp.com/pages/patient-resources



# Resources

Patient Resources about severe COPD exacerbation treatment options and Advance Care Planning

http://decisionaid.ohri.ca/docs/das/COPD.pdf

**What is advance care planning?**

- Advance care planning is planning ahead for future medical care in case you are unable (too sick) to make your own medical decisions.

**How can I plan for advance care?**

- Talk to your doctor and your family about your values and your wishes for end-of-life care.
- Use the resources and websites listed on this sheet to find more information.

**Where can I find out more about advance care planning?**

- At NYU Medical Center: NYU's Patient and Family Resource Center
  - Location: 530 First Avenue, Schwartz Health Care Building, First Floor - Room 103, New York, NY 10016
  - Phone: (212) 263-7438
  - Website: http://pfrc.med.nyu.edu
- Online:
  - www.CompassionAndSupport.org
  - www.PrepareForYourCare.org

**How can I get help on how to you talk to loved ones about end-of-life wishes?**

- Online: http://theconversationproject.org/starter-kit/intro/

**What is an advance directive?**

- An advance directive is a *legal document* that allows you to put into words your choices about end-of-life care ahead of time.
- With an advance directive, you can make your wishes known to family, friends, and health care providers ahead of time. It tells your doctor what kind of care you would like in case you are unable (too sick) to make medical decisions.

**Where can I find out more about advance directives?**

- Talk to your doctor.
- Contact the NYC Department of Health (Online: http://www.nyc.gov/html/doh/html/hca/advance-directives.shtml)
- Online: http://familydoctor.org/familydoctor/en/healthcare-management/end-of-life-issues/advance-directives-and-do-not-resuscitate-orders.printerview.all.html

**How can I get help creating an advance directive?**

- Ask your doctor.
- Call 311 and ask for Health Care Proxy forms
- Talk to a lawyer (with your family members at your side if you choose)
- Online: https://www.makingyourwishesknown.com/

Back
